# Marketing techniques today’s real challenge to help customers adopt healthy eating habits

**Published:** 2014

**Authors:** Raţiu Monica Paula, Oprescu Alina Elena

**Affiliations:** * Romanian – American University, Bucharest, Romania

**Abstract**

Today, communication with consumers and their influence towards adopting healthy eating habits must represent a priority for public health authorities in every country around the world. This paper presents the main marketing resources, which can be used to healthier eating promotion among consumers. As nowadays marketing represents a key instrument in influencing the food choices of the modern consumer, marketing techniques can be also used to promote healthier eating among consumers and reduce the impact of serious diseases.

**Keywords:** marketing techniques, customers, healthy eating habits

